# Supplementary material for: Transposable element insertions in 1000 Swedish individuals
Source: PLoS One. 2023 Jul 28;18(7):e0289346. doi: 10.1371/journal.pone.0289346 (PMC10381067; doi:10.1371/journal.pone.0289346)
Supplement: S1 File — (DOCX) [file pone.0289346.s001.docx]

Supporting Information 1


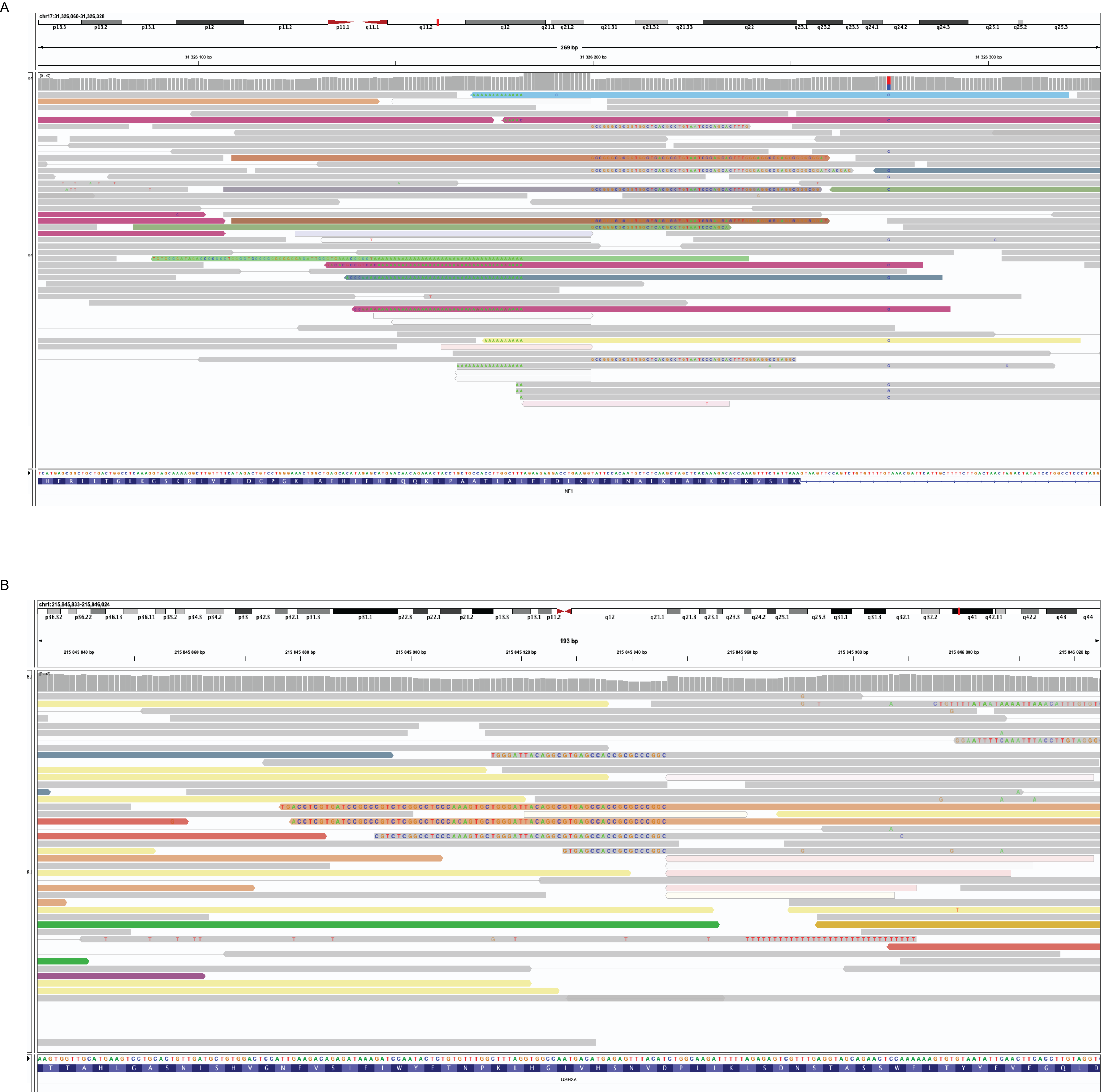


**S1 Figure 1: Integrative Genome Viewer image of (A)** *Alu* insertion in exon 37 of *NF1.* **(B)** *Alu* insertion in exon 45 of *USH2A.*

*
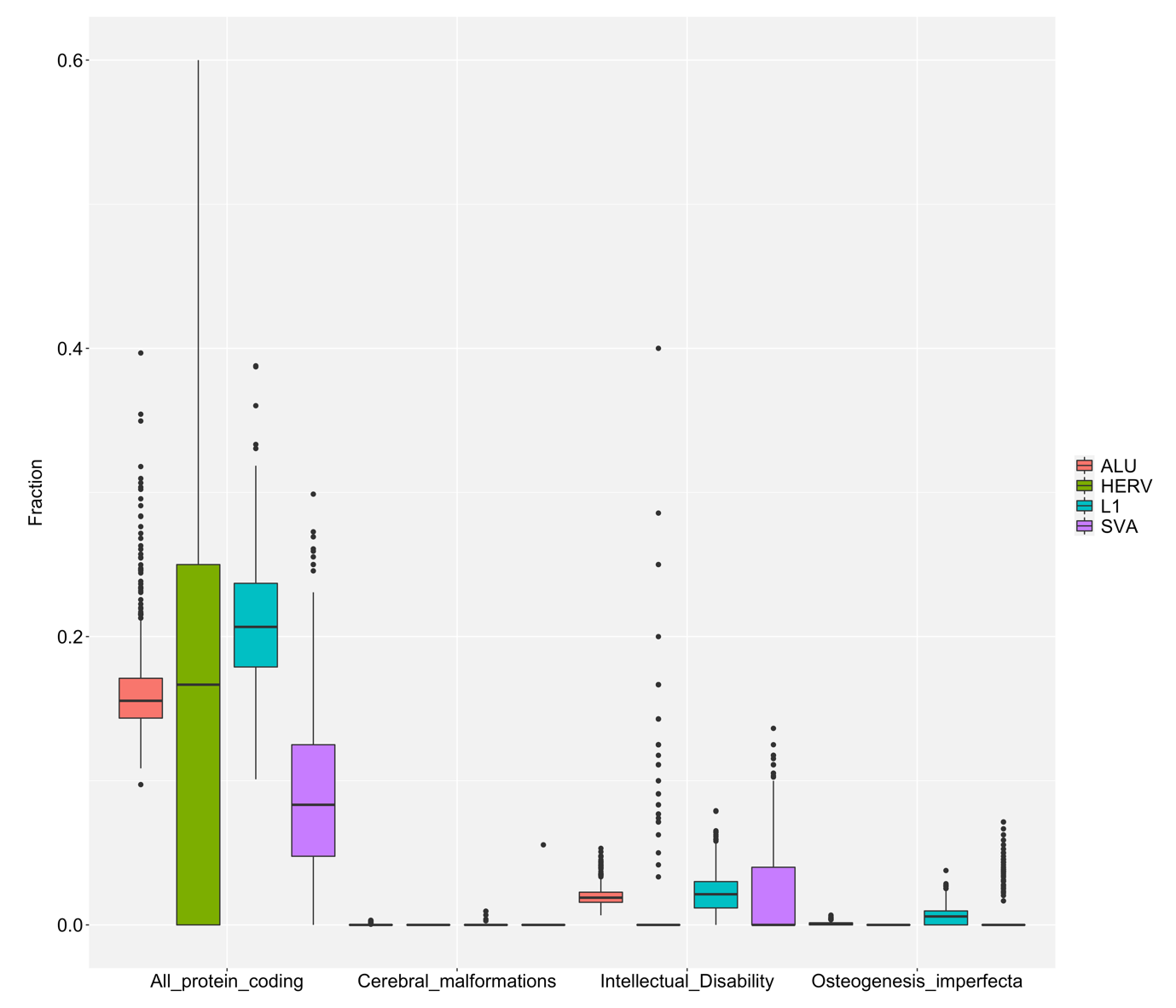
*

**S1 Figure 2: TE insertions overlapping with genes in various gene lists.** Fraction (TE insertions in protein coding gene from list/all protein coding TE insertions – per TE type) of *Alu*, HERV, L1 and SVA elements in the SweGen present in the 1KGP dataset at an allele frequency >0.01, affecting protein coding genes present in gene lists; all protein coding (n=22882), cerebral malformations (n=107), intellectual disability (n=2391), or osteogenesis perfecta (n=185).
