# Supplementary material for: Transposable element insertions in 1000 Swedish individuals
Source: PLoS One. 2023 Jul 28;18(7):e0289346. doi: 10.1371/journal.pone.0289346 (PMC10381067; doi:10.1371/journal.pone.0289346)
Supplement: Attachment — Submitted filename: Responses to Plos Genetics Reviews Questions(1).docx [file pone.0289346.s003.docx]

Responses to Reviewers Points

**Reviewer #1:** Saether et al report the re-analysis of SweGen and 1KGP sequencing data to call non-reference TE insertions using an ad hoc bioinformatic pipeline. Despite the lack of benchmarking of the sensitivity and specificity of the pipeline, the authors carried a characterization of the putative TE insertions. They show that most non-reference TE insertions calls belong to Alu, followed by L1, consistent with previous analysis performed in the 1001K population (e.g. Sudmant 2015, Nature; Cao et al 2020, Genome biol; Borges Monroy et al 2021, MobDNA). They also performed an enrichment analysis and report that exons are depleted in such non-reference TE insertions, confirming previous observations (e.g. Borges Monroy et al 2021, MobDNA) and as expected given the negative impact of such mutations. Last, they identify some putative non-reference insertions in the genome of neurofibromatosis patients. The relevance of these insertions were not studied.

As you will note from my comments below, my main issue is the lack of quality control and that I have issues to identify the novelty of the article.

**Major points**

**Comment:** Detection of TE insertion polymorphisms based on short reads is very noisy and here the authors do not provide any convincing characterization of the sensitivity and specificity of their pipeline. Validation with orthologous methods such as long reads or PCR should be provided. Also, the detection od TE insertion should be accompanied by the detection of TSD, which is the hallmarck of true transposition events.

**Answer:** We rebuilt our analysis to only include RetroSeq, a TE caller where the specificity and sensitivity has previously been benchmarked (doi: 10.1093/bioinformatics/bts697, 10.1093/bib/bbw072, 10.1186/s13100-019-0197-9). To address the potential noisy output, we perform filtering according to RetroSeqs quality flagging, as well as filtering using an in-house database consisting of 1000 Swedish and 2504 genomes from the 1000 Genomes Project. Additionally, we are able to identify two clinical cases where the caller is able to correctly identify disease causing *Alu* elements.

**Comment:** Most reported results are either not novel or else contradictory with previous literature. For instance, the contribution of the different mobile TE families to the total number of non-reference TE insertions is very well stablished, as well as their genome-wide distribution and association with gene features (Figure 1). On the other hand, the allele frequency of the filtered non-reference insertions (Figure 3) is skewed towards common variants, which contradict most studies to date showing that non-reference TE insertion are typically rare. It appears that your observations are strongly biased due to the filters you imposed.

**Answer:** The novelty of the paper has been revised, and the focus is centered around the clinical value of TE calling and analysis rather than the impact TE insertions have on genomic features. Figure 3 with TE filtration in 22 SweGen individuals in the previous paper has also been revised. A similar, but different scope is current Figure 4, with the fraction of SweGen TE insertions with a certain allele frequency in the 1KGP databases. We believe this is more relevant, and the distributions here are different, with higher fractions of rare non-reference TE insertions. Regardless, high fractions of common non-reference TE insertions can be due to bias of the caller, or simply that the TE insertion is present across all samples but cannot be found in the reference. With the large amounts of differing TE insertions across individuals, this would not be unlikely.

**Comment:** Lines 235-241. It is not clear where the data corresponding to the “80 cases with suspected Neurofibromatosis” come from nor the type of data and its quality. In the same line, it is unclear how you conclude that “27% (n=22) a pathogenic or likely pathogenic variant vas detected”. Idem with the Retinitis Pigmentosa dataset.

**Answer:** We have revised this section and clarified where the cohort comes from and why we chose these sample. The two clinical cases originated from cohorts of patients with suspected neurofibromatosis (n=80) and retinitis pigmentosa (n=54) analyzed at the Karolinska clinical genetics diagnostic laboratory. Disease-causing TE insertions had previously been suspected in these cases, but not identified by the in-house SV pipeline. Out of these 80 cases, the pathogenic or likely pathogenic variant was detected in 22 (27%).

**Other specific points:**

**Line 157**. It is not clear why you focused in SweGen... It is unclear what do you mean with “due to preprocessing of the 1KGP” you choose to focus on SweGen. You should provide a sound justification or else perform the analysis using all the data sets.

**Answer:** In the revised version we do not perform characterization of genomic features, and we have performed same analysis on both datasets.

**Line 162**: Figure 1D does not contain intronic features. In addition, could you explain how the enrichment was calculated? In relation to the genomic space covered by the features or by generating a base-composition-matched control datasets as in Sultana et al 2019, Mol Cell?

**Answer:** In the revised version we do not perform characterization of genomic features.

**Line 165.** I don’t find anywhere the information of “intronic pseudogenes”

**Answer:** In the revised version we do not perform characterization of genomic features.

**Line 167**: how is it possible that you detected 51031 Alu insertions within other Alus given that the total number of identified Alus is 30903 (line 126)?

**Answer:** In the revised version we do not perform characterization of genomic features.

**Line 181-182**. It is unclear why non-referent insertion with AF<2% were excluded. Which quality control could not be performed? Indeed I don’t find any quality control of none of the calls.

**Answer:** We have in our revised manuscript included TE insertions with all allele frequencies, except for in Figure 3, where we took all above 1%, this as we wanted to see the distributions of TE insertions that are rare, but not novel.

**Line 188**: The genes are not rare, but their TE-containing alleles.

Please, when you are referring to your calls, specify that these are "non-reference"

**Answer:** We refer to our calls as TE insertions.

**Reviewer #2:** I have reviewed the manuscript of Bilgrav Saether et al., entitled "Transposable element insertions in 1000 Swedish individuals". The authors examine the insertional polymorphism of retrotransposons in 1000 samples of the human cohort SweGen and perform a comparison with the 1000 Genomes cohort (1KGP). To this end, the authors have developed a new pipeline combining Jitterbug and RetroSeq (TE-specific programs) and Delly, a general purpose SV caller. Though extremely interesting, I think the method used need to be properly evaluated before moving forward. In particular false and true positives rates should be estimated and comparison must be performed with recently published and competing methods (see below.).

Nevertheless, and before detailing the expected revisions, I would like to emphasize two points which I found very interesting and original. The first is the interest given to self-insertion of TE (though the performance of the pipeline on this issue may need to be assessed, for example through simulation). And second the program MobileAnn which allows the authors to identify TE detected by Delly (which was not specifically developed to identify them) is a notable development.

**Main:**

**Comment:** As mentioned above, the results of the pipeline or at least a subset must be compared with at least one of MELT2 (Gardner et al., 2017 -- used by 1KGP) or xTEA (Chu et al., 2021). RetroSeq and Jitterbug are from an older generation of tools, and even though they may provide sufficient performance, several other tools, included those mentioned above have been shown to perform better (see: https://mobilednajournal.biomedcentral.com/articles/10.1186/s13100-019-0197-9 and https://academic.oup.com/bib/article/18/6/908/2562836 for example). I understand that there is a plethora of programs with the same purpose and it is impossible to compare the authors' pipeline to all of them. Conversely, it would be useful for potential users to understand the potential advantage of the authors' method if it is deemed to perform better than already existing tools. In addition, Delly is very interesting to consider, as the use of general purpose SV callers has not been extensively tested in their potential help in finding insertion polymorphisms of TE. However, results must be compared with some existing tools such as MELT2, xTEA, etc...

**Answer:** xTEA and MELT2 are great programs. However, xTEA is a pipeline, whilst MELT2 requires a license to be used in the clinic. This makes these programs challenging to include in our diagnostic structural variant pipeline. Additionally, with the alteration of the workflow, we only include RetroSeq, due to its easy implementation and running, as well as performance in earlier mentioned benchmarking studies.

**Comment:** Regarding the benchmarking of the method, the authors can make use of the HG002 sample from Genome In A Bottle it (GIAB). It could also be insightful to perform simulation in order to evaluate the performance of the pipeline in detecting accurately TE within TE.

**Answer:** Benchmarking studies of RetroSeq has been performed previously (doi: 10.1093/bioinformatics/bts697, 10.1093/bib/bbw072, 10.1186/s13100-019-0197-9). In the revised version we do not perform characterization of the genomic regions of non-reference TE insertions.

**Comment:** I did not see mention of reference insertions (i.e. those present in the reference genome but not in a given sample). MELT2 can perform this analysis for example. Reference insertions might represent 1/10 of polymorphic TE insertions in human and are generally segregating at higher frequency (> 5%). Unfortunately they are often not analyzed but can be relevant functionally (https://pubmed.ncbi.nlm.nih.gov/32075552/ for example [I am a co-author]).

**Answer:** With our scope to find non-reference TE insertions that could be relevant to disease, we believe that TEs not present in our sample is beyond the scope of our project.

**Comment:** The authors investigate the polymorphisms of human endogenous retroviruses (HERVs). This is very welcome however there are several points for which I would like to see more details. 1) Are those real insertion polymorphisms or LTR-LTR recombination events (~90% of HERVs are of the solo-LTR form following LTR-LTR recombination)? 2) Please provide the superfamily if possible (HERV-K, HERV-H and HERV-W in particular). It is expected that mainly HERV-K (HML2) would have insertion polymorphisms and some other (older) families such as HERV-W and HERV-H may have polymorphism between full-length (proviral) and solo-LTR (<https://doi.org/10.1186/s13100-018-0142-3>).

**Answer:** With focus removed from characterization of the non-reference TE insertions, we believe it is sufficient to include all subgroups of the various TE to their main group; *Alu*, HERV, L1 and SVA.

**Comment:** Though not critical, I think it would have been better to align the data to hg38 (2013) instead of hg19 (2009). The 1KGP cohort 30X short-read data has been realigned to hg38 a few years ago, so comparison is possible.

**Answer:** In our revised manuscript, all analysis is performed using hg38.

**Comment:** TE enrichments (two-sided binomial test + Bonferroni) should be described in more details for replicability.

**Answer:** The enrichment analysis is not included in the revised version.

**Comment:** Several relevant publications useful for comparison are missing (see minor for more details).

**Answer:** We have read and included relevant publications.

**Minor:**

65. xwe -> we

95. "The germline de novo transposition rate is approximately 1 in 29 births for Alu, 1 in 117 births for L1 and 1 in 206 births for SVA (10)" -- You might also want to compare and cite the results of <https://genome.cshlp.org/content/29/10/1567.full>

**Answer:** This has been included.

107. "TEs have been characterized in short read genome data (SR-GS) by the 1000 Genomes Consortium (1KGP) (4)(19)". Other recent publications includes: https://academic.oup.com/gbe/article/12/6/779/5828221?login=true (I am a co-author); https://academic.oup.com/nar/article/50/5/2493/6536893 and the preprint <https://www.biorxiv.org/content/10.1101/2022.03.25.485726v1>

**Answer:** We have read and included relevant publications.

141. Why 10% specifically? It could be interesting to compare at 5% which is often used as a low-frequency cutoff

**Answer:** A 5% cutoff has been included (Figure 3).

**Reviewer #3:** This manuscript details a study of the distribution of transposable elements insertions in a human population sample of swedish individuals. The identification of TE insertions is based on the combination of three methodological approaches, and the genomic distribution is analyzed, including a GO characterization of the genic insertions. The insertions in two clinical cases are also described in some details.

Overall, while I appreciate the importance of the general issue addressed (the role of TE variation in human populations and its eventual clinical relevance), I have several major concerns : that prevent me from recommending this paper for publication in such a generalist journal in the field as PLOS Genetics.

**Comment:** First, the manuscript remains entirely descriptive. The numbers of insertions are reported, as well as their genomic distribution, but the messages the readers are supposed to get from these analyses is not made apparent. This is especially problematic since the introduction and the whole manuscript are not framed in a way that clearly describes the state of the field, what our understanding is on these questions and where the knowledge gap lies. There are already lots of data on the distribution of TE insertions across human populations, whose patterns should be described in much more details so as to formalize a series of hypotheses that are currently lacking.

**Answer:** We have revised our message and now focus on the clinical implementation of non-reference TE insertion analysis, and want the take home message to be that TE insertion analysis should not be overlooked in clinical analysis.

**Comment:** Second, I was not entirely convinced by the detection method employed. As I understand, the in-house TE-detection pipeline relies on the combination of three different callers. The reason why developing this new pipeline was necessary is not justified in the manuscript, and the consequences of this particular choice of methods on the results are not properly evaluated beyond vague statements in the discussion. Without such a detailed evaluation, it is currently impossible to compare the results of this particular study to the previously described patterns (from the papers cited in the ms). Also, the added value of developing a swedish-specific TE insertion database as compared to a more global database should be evaluated : how many insertions would be missed, and how many insertions would be wrongly inferred ?

**Answer:** By moving away from the combination of callers to only one, we are able to compare our results with that of others applying similar methods. By comparing the SweGen samples to the databases created using the 1KGP dataset (Figure 4), we can confirm that there is an added value in having larger databases that span across multiple populations for the best possible filtration.

**Comment:** Third, I was also not convinced by the analysis of the two clinical cases used to benchmark the pipeline. First, the reason why only two individuals were used needs to be stated clearly, when one would like to see a much broader confirmatory set. Second, I could not understand why the confirmation step focuses only on these two specific TE insertions. The manuscript says that these two insertions are « pathogenic », but no evidence of this is provided in the text. I am not a clinical geneticist myself, so may be getting this wrong, but as I understand at this stage you are only observing correlations, which is not sufficient to establish that they are « disease-causing » (or at least this should be explained for the PLOS Genetics audience). In addition, and more importantly, a proper test of the pipeline would compare also the whole set of predicted TE insertions in these two individuals, reporting the rate of true and false positives, as well as the rate of false negatives,

not just the capacity to pinpoint these two particular mutations.

**Answer:** The two individuals identified in our routine clinical analysis. We therefore analyzed these to confirm that our workflow was able to identify these clinical cases. We have updated the description of these samples to be clearer. We have also included a figure describing amounts of insertions throughout the workflow (Figure 1) to meet these concerns.

**Comment:** Finally, I was not convinced by the enrichment analysis presented for the TE insertions. The results show a specific enrichment of genes involved in brain-related functions among the Alu and HERV insertions. Beyond the fact that it is not clear whether this relates to an insertion bias or to preferential retention of these TEs into these genes, interpreting these results would absolutely require comparing them to previous observations in humans : is this classically observed and so was already fully expected, or is this new (and if so, how should that be interpreted : a bias of the detection method or a specificity of the swedish population ?)

**Answer:** Instead of focusing on the enrichment of genes affected by TE insertions, we apply gene lists commonly used in a clinical setting (Figure 5). We report the fraction of all protein coding non-reference TE insertions affecting genes found in the gene list. We believe this to be a better representation of the distribution of which non-reference TE insertions affect genes associated to diseases.

**Reviewer #4:** In this manuscript, Eisfeldt and colleagues identify insertional polymorphisms mediated by transposable elements (TE) in 1000 Swedish individuals. This could represent a useful resource to identify TE insertions responsible for genetic diseases by offering a catalogue of TEs and their allele frequency in the Swedish population. However, the current study does not provide sufficient quality control for the datasets generated, and suffers from too many flaws. Therefore, I cannot support publication in PLoS Genetics.

**Major points:**

**Comment:** As the TE calling pipeline is new, careful validation and quality controls should be provided. The number of insertions detected in each individual suggests a very high false positive detection rate. Without being exhaustive, such controls could include TE calling for golden standard genomes analyzed by other TE calling pipelines, comparison of results with MELT, xTea, cross-validation using existing TE databases, etc. As far as understand, the only effort to reduce the rate of false positives is to eliminate insertions below an arbitrary threshold of allele frequency. However, this does not eliminate systematic errors, and much of the presented results appear to include an unfiltered dataset (a paragraph entitled ‘testing of pipeline and database filtration’ is introduced in the middle of the paper). The authors should consider to first validate the pipeline, then to define an appropriate filtering scheme, and finally to provide a consolidated high-confidence dataset, on

which they can then perform subsequent characterization.

**Answer:** See above comment for reviewer #2. Retroseq has reported a false discovery rate of 7.7%, 6.7% false negative rate, a sensitivity of 75% and a precision of >95% as has previously been reported by benchmarking studies where Retroseq has been used (doi: Ho10.1093/bioinformatics/bts697, 10.1093/bib/bbw072, 10.1186/s13100-019-0197-9).

**Comment:** Once a high-confidence dataset is obtained, the list of TE insertions should be made available as a supplementary table to permit reuse, and not only in an online database (at least in an aggregated form if a list of TE for each individual raises privacy issues), as it appears there is no specific way to specifically select TE-mediated variants through the online portal.

**Answer:** In the online portal (https://swefreq.nbis.se/), databases in vcf format can be downloaded for each TE type. The file is a multi-sample vcf and contain all TEs per individual.

**Comment:** Several of the biological observations are likely artefacts. First, the finding that TEs are enriched for self-insertions probably reflects that the TE annotation file used for enrichment calculation contains the polymorphic events themselves (i.e. many of the “Swedish alleles” are already present in the 1KGP dataset). Second, apparent enrichment in genes related to neuronal functions or pathologies is a known artefact of GO analysis as these genes are longer. They authors should consider using softwares that take into account gene length when performing this analysis (such as GREAT). Thus, the link with neurodevelopment and disease is poorly supported. Last, the inclusion of histone marks for enrichment analyses is difficult to interpret as these marks are highly cell-type dependent.

**Answer:** We have in the revised version moved away from this analysis. Instead, we summarize the fraction of non-reference insertions in protein coding genes present in chosen gene lists (Figure 5).

**Comment:** It seems that the called dataset only represents non-reference TE insertions. It would be important to look for reference TE insertions absent in the samples (annotated as deletions in the 1KGP), and to generate a genotyping of the samples (homozygotes empty, homozygotes filled, heterozygotes).

**Answer:** As mentioned above, we believe looking for reference TEs missing from the samples is beyond the scope of our analysis, but could be worthwhile in a larger study with focus on population characterization of TEs.

**Comment:** Among population-based analyses, the authors may consider including a PCA of the 1000 Swedish genomes together with the 1KGP projects datasets to verify that the population structure follows the known genetic history of the Swedish population.

**Answer:** the SweGen samples are collected from various regions in Sweden (<https://swefreq.nbis.se/>) and represent Swedish genomic variability.

**Comment:** As for patient analysis, the benefit of using the 1000 Swedish genomes vs 1KGP datasets vs both, to filter common insertions should be demonstrated.

**Answer:** We address this issue in Figure 4, with fractions of SweGen TE insertions with a certain allele frequency in the databases 1KGP databases

**Comment:** The wording is imprecise, and the manuscript shows a general lack of proofreading. Below is a non-exhaustive list:

a. P5, l88: ‘transposase’: L1 does not encode any transposase

b. P5, l89: ‘They encode an RNA II promoter that produces proteins’ is a total biological non-sense, and should be replaced by something like “They are transcribed by an RNA-Polymerase II promoter and encode proteins …”

c. P.9 and Fig. 1, the terms ‘genomic consequences’ or ‘implications’ are very much overstatements as the authors only looked at association with genomic features.

d. P.6, l114: ‘we find that TEs (…) are vital for genome variability and evolution’. I do not see TE being vital for anything in the presented data.

e. Fig.4 legend does not match the figure and appears to be incomplete. The inheritance pattern of the diseases should be specified, as well as the second allele mutation if the hearing loss/retinitis pigmentosa is recessive.

f. RT should not be used as an abbreviation for ‘retrotransposon’ as it is commonly used for ‘reverse transcriptase’.

g. The ‘nuclear’ of long/short-interspersed (nuclear) elements is optional but should be consistent in the text (the N of SINEs and LINEs can come from INterspersed).

h. Fig. 1D, what is the metrics shown by the red-blue scale?

i. Fig. 3 does not show any ‘filtration’ as stated in the legend, and it is unclear what the ‘frequency’ represents. I assume it is not the allele frequency.

j. Typos: p. 3, l44, diseas->disease; p.4 l65, xwe > we; p.14 l238, vs>was

**Answer:** We have addressed these issues in our revised manuscript.

--------------------
