# Supplementary material for: Transposable element insertions in 1000 Swedish individuals
Source: PLoS One. 2023 Jul 28;18(7):e0289346. doi: 10.1371/journal.pone.0289346 (PMC10381067; doi:10.1371/journal.pone.0289346)
Supplement: Attachment — Submitted filename: Review PONE D 23 00875.docx [file pone.0289346.s004.docx]

**Review**

The manuscript was aimed to analyze transposable element insertions in 1000 Swedish individuals and they create a population specific TE insertion data base. They also tried to evaluate the TE insertion workflow on two clinical cases where pathogenic TE insertion were suspected. There are some interesting findings presented, however I believe the manuscript is not currently of sufficient quality to be considered suitable for publication. I have set out my reservations below.

**Major Concerns:**

I find that the analyzes are unreliable because we cannot rely on references to have insertions without resorting to statistical tests. It is better to use the Kmeans method. Furthermore, the RetroSeq tool used in this study is not specific to insertion and is based on orthology. Furthermore, given that the authors analyzed short reads, it is therefore essential to verify the insertions found by PCR or to validate them using another tool.

**Minor Concerns :**

**1/ Keywords**: mobile elements are synonym to transposable elements.

Keep one of the two keywords.

**2/ Introduction :**

Line 42-43: “The reasons for this could be due to the specific disease-causing gene not being described or that the underlying cause is not (mono)genetic”. It could also be due to epigenetic factors.

Line 55: The sentence « TE are DNA sequences that compose around 50% of the human genome [6].TE are DNA sequences that can change their position in the genome should be reformulated.” Should be replaced by« TE are DNA sequences that compose around 50% of the human genome that can change their position in the genome [6].”

Line 56-58: authors have to precise that class II elements transpose via DNA with cut paste mechanism (which may explain the 2% proportion) whereas class I transpose with copy paste mechanism.
